# The relationship between morphology and behavior in mixed‐species flocks of island birds

**DOI:** 10.1002/ece3.6714

**Published:** 2020-09-25

**Authors:** Brian C. Weeks, Shahid Naeem, Benjamin M. Winger, Joel Cracraft

**Affiliations:** ^1^ School for Environment and Sustainability University of Michigan Ann Arbor MI USA; ^2^ Department of Ecology, Evolution and Environmental Biology Columbia University New York NY USA; ^3^ Department of Ornithology American Museum of Natural History New York NY USA; ^4^ Museum of Zoology and Department of Ecology and Evolutionary Biology University of Michigan Ann Arbor MI USA

**Keywords:** behavioral ecology, functional traits, Melanesia, mixed‐species flocks, morphology, ornithology

## Abstract

Understanding how co‐occurring species divide ecological space is a central issue in ecology. Functional traits have the potential to serve as a means for quantitatively assessing niche partitioning by different species based on their ecological attributes, such as morphology, behavior, or trophic habit. This enables testing ecological and evolutionary questions using functional traits at spatio‐temporal scales that are not feasible using traditional field methods. Both rapid evolutionary change and inter‐ and intraspecific competition, however, may limit the utility of morphological functional traits as indicators of how niches are partitioned. To address how behavior and morphology interact, we quantified foraging behavior of mixed‐species flocks of birds in the Solomon Islands to test whether behavior and morphology are correlated in these flocks. We find that foraging behavior is significantly correlated with morphological traits (*p* = .05), but this correlation breaks down after correcting for phylogenetic relatedness (*p* = .66). These results suggest that there are consistent correlations between aspects of behavior and morphology at large taxonomic scales (e.g., across genera), but the relationship between behavior and morphology depends largely on among‐clade differences and may be idiosyncratic at shallower scales (e.g., within genera). As a result, general relationships between behaviors and morphology may not be applicable when comparing close relatives.

## Graphical abstract

Understanding how co‐occurring species divide ecological space is a central issue in ecology. We demonstrate that morphology may reflect behavioral niche partitioning at large scales, but among islands in the Solomon Archipelago, shifts in avian morphology and behavior are decoupled. General relationships between behaviors and morphology may not be applicable when comparing close relatives.

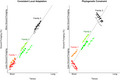

## 1 INTRODUCTION

A central focus of ecology is to understand how and why species coexist through the partitioning of resources. Classic studies of birds have tested niche partitioning hypotheses using detailed observations of behavior and various methods of quantifying ecological space to characterize the ecological niches of species (Grinnell, 1917; MacArthur, 1958). Functional traits have also been used to elucidate community structure through the analysis of partitioning of niche space (Ricklefs & Travis, 1980). Trait‐based methods for determining community structure assume that morphological features of species reflect not only their local adaptations to the environment but also their biotic interactions. Traits such as tarsus length, bill dimensions, and wing morphology have been associated with complex foraging behaviors in various contexts, including within clades (Botero‐Delgadillo & Bayly, 2012), across taxonomic groups within a community (Miles & Ricklefs, 1984), across major adaptive radiations (Fitzpatrick, 1985), and across all birds (Pigot et al., 2020). These studies reflect a general consensus: Bird morphology is broadly correlated with foraging behavior, suggesting that morphology can be used as a proxy or indicator of foraging behavior. As such, functional traits are often used as surrogates for ecological differences across spatial and temporal scales that are beyond the scope of direct observation (e.g., Weeks & Claramunt, 2014; Weeks, Gregory, & Naeem, 2016).

Despite widespread use of morphology as a proxy for behavior, there are examples of behavior and morphology being decoupled. For example, a “fear of flying” may result in behavioral flightlessness in some birds (Diamond, 1981), such as in some island endemic *Zosterops* bird species (Moyle, Filardi, Smith, & Diamond, 2009) that have retained their morphological flight apparatus but have adapted their behavior to greatly reduce their flight. More broadly, behavioral traits tend to be more evolutionarily flexible than morphological traits (Blomberg, Garland, & Ives, 2003). Together, these findings suggest that in some circumstances behavioral changes might facilitate novel foraging behavior without requiring morphological adaptation, potentially limiting the utility of morphology as a proxy for behavioral differences (Wiens & Rotenberry, 1980).

To explore the extent to which morphology predicts behaviors associated with niche partitioning, we take advantage of mixed‐species foraging flocks in the Solomon Islands. Birds form mixed‐species flocks in virtually all habitats, and they have been documented on every continent. Participation in flocks can result in improved predator avoidance and improved foraging efficiency, though it can also be ecologically costly (reviewed in Sridhar, Beauchamp, & Shanker, 2009).

The benefits of mixed‐species flocking can result from the association with individuals of the same or similar species (i.e., supplementary benefits), or complementary interactions among species in which interspecific differences provide benefits to group members (Goodale et al., 2020). Due to the intensity and complexity of interspecific interactions in mixed‐species flocks, constituent species may be exposed to novel biotic selective pressures (Harrison & Whitehouse, 2011). When species are in mixed‐species flocks they may change their feeding rates (Hino, 2000), the location of their foraging within the canopy (Farine & Milburn, 2013; Zou, Chen, Yang, & Fellowes, 2011), and their method of prey capture to take advantage of interspecific complementarity (Satischandra, Kudavidanage, Kotagama, & Goodale, 2007). Further, these interactions may vary across the landscape, stimulating rapid behavioral adaptation, with participant species likely exhibiting high behavioral flexibility across space (Knowlton & Graham, 2011). This behavioral flexibility may disassociate morphology and species' behaviors within mixed‐species flocks.

In Northern Melanesia, qualitative descriptions of mixed‐species foraging flocks have characterized them as regular fixtures of the avifauna, and important components of the natural histories of many species (Cowles & Uy, 2019; Diamond, 1975a; Dutson, 2011; Kratter, Steadman, Smith, Filardi, & Webb, 2001; Weeks et al., 2017). In New Guinea, mixed‐species flocks are found that appear to have both elements of the supplementary and complementary models of Goodale et al. (2020). Flocks centered around the Papuan babbler (*Garritornis isidorei*), for example, include constituent species that mimic the babbler's plumage and call (Bell, 1983; Diamond, 1987). This convergence in plumage has been attributed in part to a supplementary benefit: enhanced confusion of predators (Diamond, 1987; Prum, 2014). In other New Guinea flocks, centered around *Gerygone* spp., complementarity appears to be a more dominant force. For example, in the *Gerygone*‐based flocks—also called “small insectivore alliances”—the hawking species (e.g., *Rhipidura* spp.) were observed to follow the gregarious *Gerygone* species, apparently preying on disturbed insects (Bell, 1983). These examples illustrate that behavioral plasticity is a commonality across flocks, with constituent species altering their behaviors upon joining mixed‐species flocks (Bell, 1983).

**FIGURE 1:**
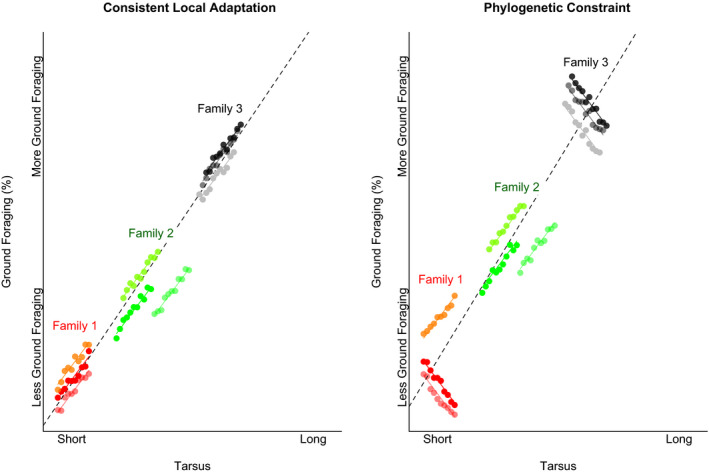
Consistent local adaptation or phylogenetic variation. General relationships between morphology and behavior (e.g., the positive relationship between tarsus length and proportion of foraging that is done on the ground; dashed black line) can have different relationships with smaller scale patterns. First, the large‐scale relationship may be consistent with the relationship between morphology and behavior within smaller taxonomic groups (a; in which tarsus and percent of foraging are positively correlated within genera—indicated by color—and families). Second, while there may be a general relationship between morphology and behavior, it is possible that within smaller taxonomic groups, this relationship is inconsistent (b). This signal of evolutionary history may take the form of local adaptations in which the relationship between morphology and behavior within genera does not match the general relationship

**FIGURE 2:**
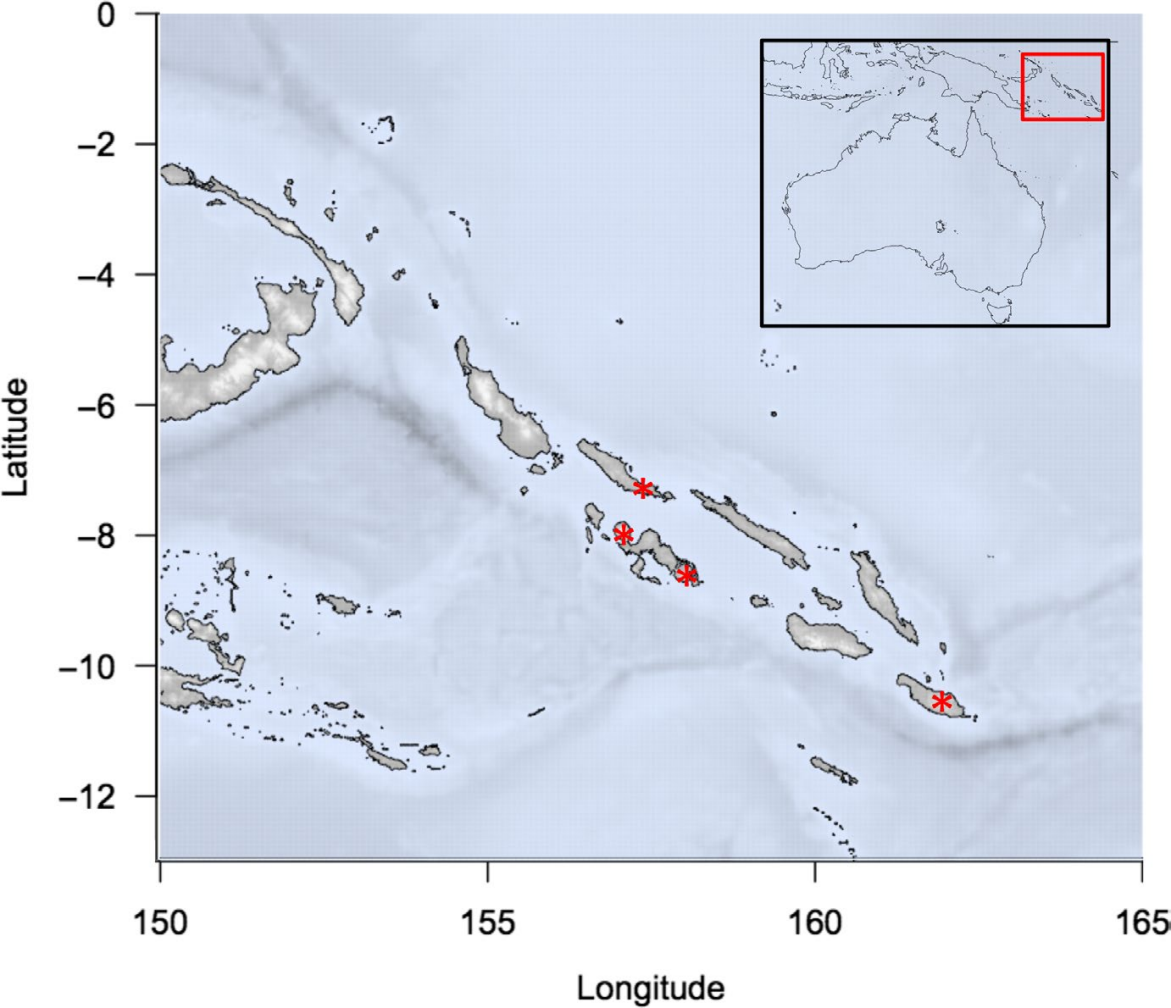
Sampling locations across the Solomon Archipelago. Mixed‐species foraging flocks were observed on Choiseul, Kolombangara, Vangunu, and Makira (from west to east, marked with asterisks)

In addition to flocking‐associated shifts in behavior, colonization of insular systems exposes species to novel biotic and abiotic pressures, which may lead to rapid adaptation. As such, in assessing mixed‐species foraging flocks across the Solomon Islands, we are examining a system that is characterized by biotic, abiotic, and historical conditions that are expected to be ideal for stimulating rapid behavioral adaptation, potentially decoupling morphology and behavior. Additionally, while behavior and morphology are expected to be correlated to some extent, the degree to which those relationships are recapitulated across taxonomic scales remains unclear (Figure 1). For example, relative tarsus length may be correlated with the degree to which con‐familial species forage on the ground (Fitzpatrick, 1985). Yet within a genus, the relative tarsus length may not be similarly correlated with variation in differential rates of ground foraging (Figure 1). Here, we quantify foraging behavior for mixed‐species foraging flocks from four islands in the Solomon Archipelago: Kolombangara, Choiseul, Makira, and Vangunu (Table 1; Figure 2) to investigate how morphology and foraging behavior are related. By comparing flock behavior and morphology in similar sets of species replicated across multiple islands, we provide a framework to test the hypothesis that morphology and foraging behavior are correlated in species that participate in mixed‐species foraging flocks.

**TABLE 1:** Flock species composition across islands

Genus	Island			
	Choiseul	Kolombangara	Vangunu	Makira
*Zosterops*	*Z. metcalfii exiguus* ^a^	*Z. kulambangrae kulambangrae* ^a^	*Z. kulambangrae kulambangrae* ^a^	*Z. ugiensis ugiensis*
*Monarcha*	*M. castaneiventris castaneiventris*	*M. richardsii* ^b^	*M. richardsii*	*M. castaneiventris megarhynchus*
*Symposiachrus*	*S. barbatus barbatus*	*S. browni browni*	*S. browni browni*	*S. vidua vidua* ^a^
*Rhipidura*	—	*R. rufifrons granti*	*R. rufifrons granti*	*R. rufifrons russata*
	*R. cockerelli interposita*	*R. cockerelli albina*	*R. cockerelli albina*	—
*Myiagra*	*M. ferrocyanea ferrocyanea*	*M. ferrocyanea feminina*	*M. ferrocyanea feminina*	*M. cervinicauda*
*Pachycephala*	*P. orioloides orioloides*	*P. orioloides centralis*	*P. orioloides centralis*	*P. orioloides cristophori*
*Myzomela*	*M. lafargei*	*M. eichhorni eichhorni*	*M. eichhorni eichhorni*	*M. tristrami*
*Micropsitta*	*M. finschii nanina*	*M. finschii tristrami*	*M. finschii tristrami*	*M. finschii finschii*
*Meliarchus*	—	—	—	*Meliarchus sclateri^2^*
*Dicaeum*	*D. aeneum aeneum*	—	—	—
*Aplonis*	*A. grandis grandis*			*A. dichroa*
*Coracina*	*C. tenuirostris saturatior*	*C. tenuirostris saturatior*	*C. tenuirostris saturatior*	*C. salamonis*
		*C. lineata ombriosa*		
*Phylloscopus*		*P. poliocephalus pallescens*		*P. poliocephalus makirensis*
*Dicrurus*				*D. bracteatus*

Note

Dominant species are those species that were most often observed to be the most vocal and apparent leaders of the movements of the flock. Taxonomy follows (Dutson, 2011).

^a^ Dominant nuclear species.

^b^ Second most abundant/core species, noted if it was occasionally the nuclear species.

## 2 METHODS

### 2.1 Flock characterization

Mixed‐species flock descriptions and foraging data are based on targeted flock observations by BCW over the course of two field seasons (June 6–July 24, 2012 and June 6–July 9, 2016). The limitations of observing birds in the rainforest canopy precluded collection of comprehensive lists of constituent species in each flock or precise estimates of relative abundances within flocks, but by pooling across all observations at a locality we have developed qualitative descriptions of flock composition for each island (Table 1). The behaviors of the five most consistent genera from the flocks are analyzed quantitatively. Whereas across islands the core of the flocks was consistently comprised of the same genera, the interisland taxonomic variation within genera varied slightly. All genera had the same taxon on Kolombangara and Vangunu, but in some cases, the variation on Choiseul and Makira was at the sub‐specific level. For other genera, the taxa on Choiseul and Makira are classified as separate species (Table 1).

### 2.2 Foraging behavior

On each island, BCW walked along ridgelines over the full course of each day of observations, and behavioral data were collected whenever a flock was encountered. These observations were collected in low to mid‐elevation forest, which varied in absolute elevation from island to island, but was considered to be the forest below stunted montane forest, a readily apparent transition on all islands. Observations were only made in forest that had not been recently impacted by humans. Anecdotal evidence suggests that any abandoned village sites that were present within the study areas likely resulted in minimal impact restricted to ridges. Further, these villages were likely abandoned when communities moved to the coasts during conversion to Catholicism, which happened in three waves 1845–1855, 1898–1942, and 1946–1966 (Laracy, 1969). The lone exception to this was the Choiseul site, which had a village site nearby that has been effectively uninhabited since 1960–1980 (a date estimated by local landowners), but where there were still signs of past human habitation.

**FIGURE 3:**
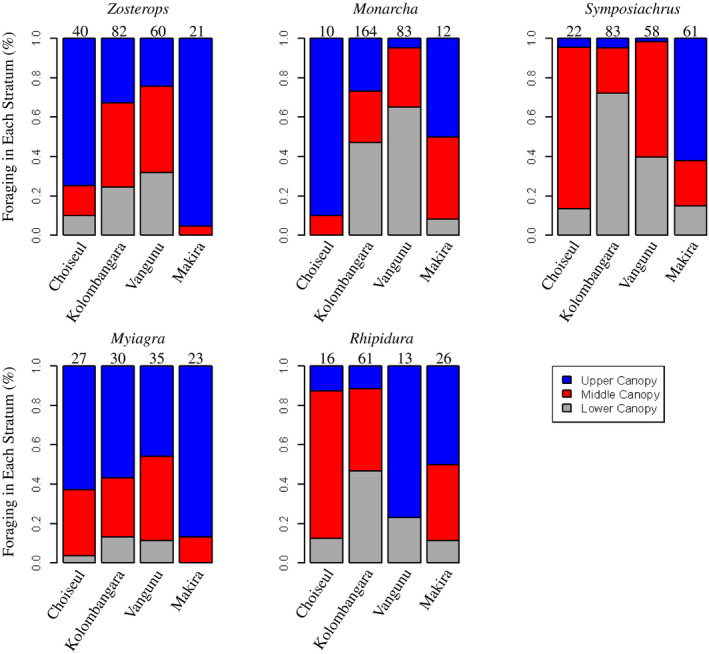
Distributions of foraging moves through the canopy. For all taxa, there is quite a bit of variation among islands in the location within the canopy where foraging takes place. Each color represents the proportion of the total foraging behavior for each taxon that occurred on an island. These data are from 927 foraging observations across the islands; sample sizes for each taxon on each island are noted at the top of each column. These interisland differences represent shifts in foraging within a taxon—between Kolombangara and Vangunu—or differences among taxa (i.e., subspecies or species; Table 1) across the other islands

**FIGURE 4:**
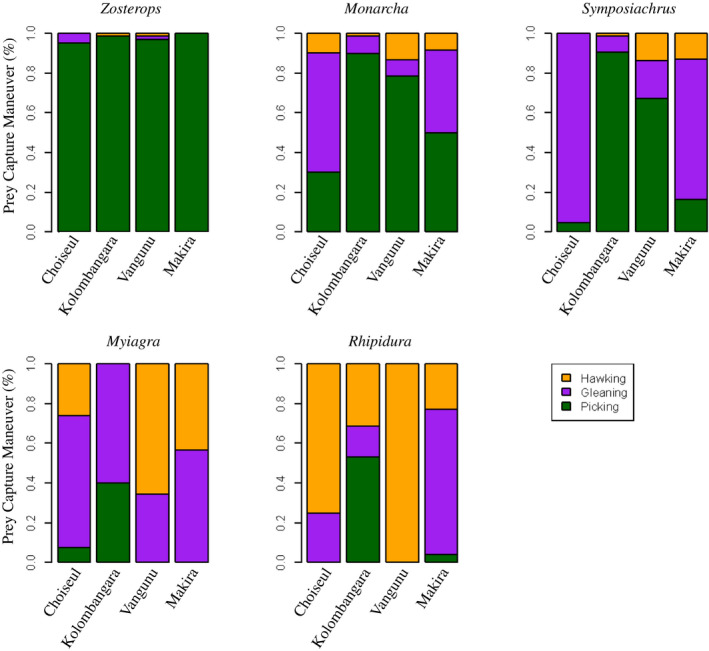
Proportion of foraging maneuver type across islands. Within genera, there are clearly differences in how much foraging maneuver type shifts among islands. Colors correspond to the proportion of total foraging effort comprised of each foraging maneuver for each taxon on each island. Sample sizes match those of Figure 3

Upon encountering a flock, any foraging maneuver made by an individual was noted, and the elevation and time of encounter were recorded. Foraging maneuvers were characterized based on whether they occurred in the lower, middle, or upper stratum of the canopy (Figure 3), and the type of move that was made: picking (capturing food while perched or hopping along a branch), gleaning (capturing food from a substrate while flying), or hawking (capturing food on the wing, in midair; Figure 4) *sensu* Holmes, Bonney, and Pacala (1979). It was not always possible to determine if the same individual was being observed making multiple moves, but we do not expect this was a frequent occurrence. Single flocks frequently yielded a limited number of observations, with an average of 2.86 foraging observations of a species in each flock. Further, when multiple moves were made by single individuals in other systems, those moves were no more correlated than moves by multiple individuals of the same species (Holmes et al., 1979).

Reciprocal averaging (RA) was used to characterize foraging behavior. RA is an ordination method that can provide a statistical representation of ecological space, while simultaneously placing behavioral data within that space, and it is appropriate for use with categorical variables like our foraging data. This approach to characterizing foraging behavior has been used in previous studies to quantitatively describe foraging behavior for subsequent correlation with morphological data (Botero‐Delgadillo & Bayly, 2012; Miles & Ricklefs, 1984). We used RA to ordinate both the types of foraging moves employed and the distribution of those moves through the canopy strata for each taxon on each island (following Miles & Ricklefs, 1984). Reciprocal averaging was conducted using the detrended correspondence analysis and basic reciprocal averaging (“decorana”) function in the Vegan package (Oksanen et al., 2019), implemented in R (R Core Team, 2018).

For tests of the correlation between morphology and behavior, species scores on RA axes were used to represent the behavioral data. While there is no standard way to determine how many axes should be used to characterize an RA ordination, we took the approach that all axes would be used until there was a significant change in the magnitude of the eigenvalue for an axis.

### 2.3 Morphological data

To characterize morphology, we measured at least three adult male specimens of each species from each island, when available, at the American Museum of Natural History (for a mean of 4.3 specimens per taxon on each island, and range of 2–10, for a total of 95 specimens; Appendix S1). For each specimen, we measured wing length (length from the carpal joint to the tip of the longest primary), length from the carpal joint to the tip of the first secondary feather, tarsus length, toe length, tail length, bill width at the anterior edge of the nares, bill depth at the anterior edge of the nares, and bill length from the anterior edge of the nares to the tip of the bill. To characterize the variation in each trait within each population, we calculated the coefficient of variation (CV) for each trait for each taxon on each island. Because climate change can cause temporal shifts in morphology, had the specimens been collected at various times, their functional traits may have been shaped by different abiotic conditions, potentially adding noise to our data. However, the vast majority of these specimens were collected during a single expedition in the early 1900s. While climate change‐induced morphological shifts may have occurred over the past century, there is some evidence that warming can result in similar shifts in functional traits across ecologically diverse groups of birds (Weeks et al., 2020), so we do not expect climate change‐induced shifts in morphology to impact our results. Morphology was characterized using a principle components analysis (PCA) based on: the hand‐wing index (HWI; Claramunt, Derryberry, Remsen, & Brumfield, 2012), the ratio of tarsus to wing length, the ratio of tail to wing length, bill volume (approximated as the product of bill length, bill depth, and bill width), and middle toe length. All variables included in the PCA were first log‐transformed, following Miles and Ricklefs (1984); ratios to wing length were used to control for body size. These traits were selected because they have been correlated to behavior in birds across a range of systems (Botero‐Delgadillo & Bayly, 2012; Fitzpatrick, 1985; Miles & Ricklefs, 1984). Ordination was conducted using the “prcomp” function in the Stats package implemented in R (R Core Team, 2018). The PCA was conducted using all individuals, and each species' morphology was characterized as the means of the individual scores on each PCA axis (i.e., the scores of all individuals of a taxon on an island were averaged for each PCA axis). To estimate the correlation between morphology and behavior, a PCA was performed to ordinate all of the morphology data together; the principal component scores from this PCA were then used to represent morphology in the canonical correlation analysis linking morphology and behavior. We kept all of the axes needed to explain at least 90% of the variance in the data.

To test how distinct the morphologies of the taxa are across the islands, we used the k‐means lazy‐learning classification algorithm, implemented with the “knn” function in the class package in R (R Core Team, 2018; Venables & Ripley, 2002). First, we divided the data, putting 1/3 of the specimens into a “training” dataset and the remaining 2/3 of the specimens into a “testing” dataset. We then predicted the species and subspecies identity of each specimen in the testing dataset, based on morphology. To do this, for each testing data point, we identified the three nearest neighbors in morphological space (using Euclidean distance) in the training dataset, and assigned the species identity of the majority of those neighbors to the test data point. Whenever there was not a majority, species identity was assigned at random from the set of identities of the three nearest neighbors. We then compared our predicted identity for each testing data point to the actual identity of the point and calculated the percentage of correct classifications. Because of the small sample size, we repeated the analysis 1,000 times, randomly assigning the data to the training and testing datasets each time. We report the mean percent of correct classifications, and the standard deviation of the 1,000 random assignments.

In addition to the multivariate ordination‐based characterization of morphology, we examined two morphological traits individually: relative tarsus length (tarsus length/wing length) and the pointedness of the bill (which we characterized as bill length/bill width). These traits have been associated with the degree to which species capture prey on the ground (Fitzpatrick, 1985).

### 2.4 Correlating morphology and behavior

To test whether morphology and foraging behavior were correlated, we used canonical correlation analysis (CCA) and phylogenetic canonical correlation analysis (pCCA; Revell & Harrison, 2008) based on the mean morphology of each taxon on each island (summarized using a PCA, as described above) and the foraging behavior of each taxon on each island (ordinated with RA, as described above). CCA allows for the correlation of multidimensional data (e.g., PCA and RA results); significance of the correlation was assessed using an F‐distribution of Pillai's trace (as implemented in the canonical correlation analysis function “CCorA” in the vegan package in R (Oksanen et al., 2019; R Core Team, 2018)). Phylogenetic relatedness was controlled for in the pCCA using the “phyl.cca” function in the phytools R package (Revell, 2012). We obtained a phylogenetic tree for use in the pCCA by downloading 1,000 trees from the posterior distribution of a phylogeny for the birds of the world (Jetz, Thomas, Joy, Hartmann, & Mooers, 2012) and creating a consensus tree using DendroPy (Sukumaran & Holder, 2010) following Rubolini, Liker, Garamszegi, Møller, and Saino (2015).

To test whether relative tarsus length and bill pointedness predict the proportion of non‐aerial foraging, we regressed the proportion of picking foraging maneuvers onto these variables. We explored the impact of phylogenetic relatedness on these relationships by incorporating a variance–covariance matrix based on the phylogeny and a Brownian motion model of trait evolution, modeled using a generalized least squares approach implemented in the nlme package (Pinherio, Bates, DebRoy, & Sarkar, 2013).

## 3 RESULTS

### 3.1 Flock composition and foraging behavior

A total of 927 foraging observations were collected across the four islands (420 observations from Kolombangara, 249 from Vangunu, 115 from Choiseul, and 143 from Makira; Appendix S2). There was not a clear break in the eigenvalues of the RA axes, which ranged from 0.66 to 0.26, with similar differences between all axes, so the scores for all four RA axes were used in the CCA.

Across the four islands, the same genera formed the nucleus of the mixed‐species flocks, though the species compositions and relative abundances within flocks varied (Table 1). Additionally, across islands, there were varying degrees of behavioral change within genera, both in the location within the canopy in which foraging took place (Figure 3) and the type of foraging maneuver employed to capture prey (Figure 4). We do not find evidence that differences in sample sizes across islands are driving interisland shifts in behavior (Appendix S3).

### 3.2 Morphology

**FIGURE 5:**
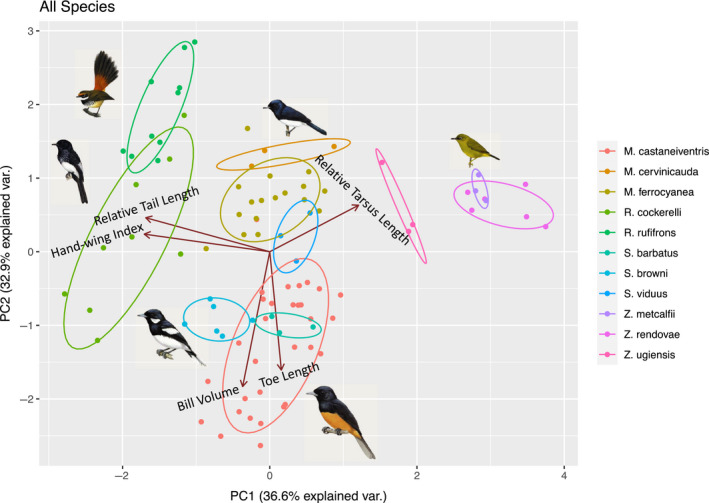
Morphospace for the mixed‐species flocks. The morphologies of the flocking species range along PC1 from longer‐tailed efficient fliers (*Rhipidura*), to intermediate morphologies (Monarchidae*)*, to longer‐legged (*Zosterops)*; this transition is accompanied by a shift in foraging behavior from largely hawking, to intermediate, to almost exclusively picking (Figure 4). PC2 largely separates the Monarchidae by body size, with the smaller *Myiagra* foraging on the wing and the larger *Monarcha* doing more picking. Each taxon is a different color. Ellipses are normal data probability ellipses, using a normal probability of 68%. Loadings are represented by the arrows, scaled by each variable's loading on PC1 and PC2. Representatives of each genus are placed in proximity to their constituent taxa in the PCA

**FIGURE 6:**
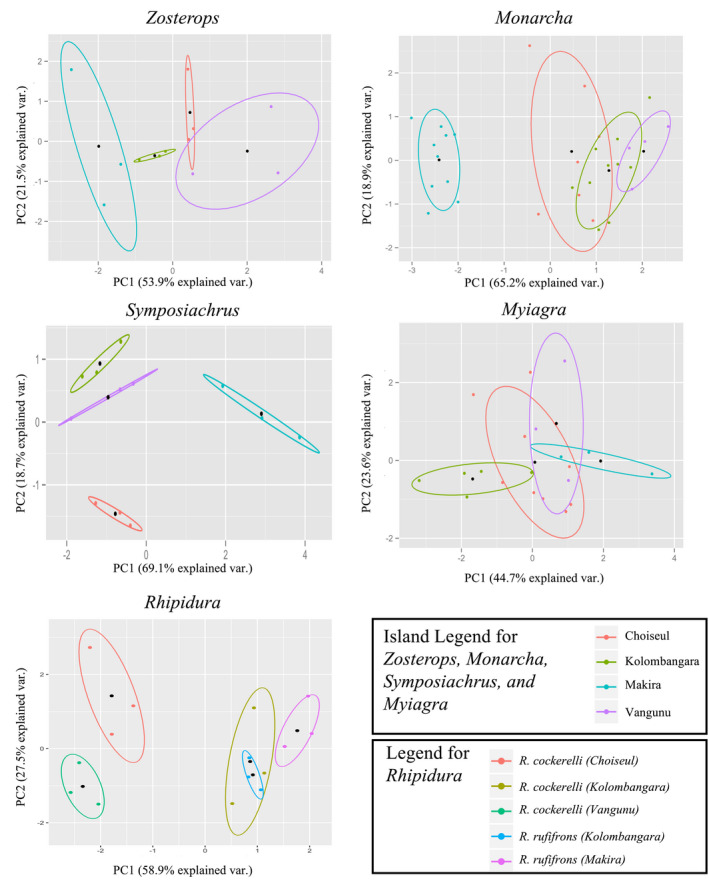
Morphological differentiation within genera across islands. Within genera, there are different degrees of morphological differentiation among islands, and these differences are not always associated with taxonomic relatedness. For example, the populations on Kolombangara and Vangunu are always the same taxon, but are not always the nearest in morphological space. Ellipses are included, showing the normal data probability distribution (68%); black dots have been added for each group, showing the mean scores for each group

We measured eight morphometric variables on 95 specimens distributed across the four islands (Appendix S1). There was limited trait variation among individuals of each population: with mean CVs across all populations for wing length = 2.97, secondary length = 2.79, tarsus = 2.38, bill height = 6.09, bill length = 3.32, bill width = 5.2, toe length = 3.15, and tail length = 2.25. The first four axes of the PCA explained 95% of the variance in morphology. Average PCA scores on these axes were calculated for each taxon on each island and used in the CCA and pCCA (*n* = 21; Table 2; Figure 5). Surprisingly, rather than PC1 reflecting size—a common assumption (Jolicoeur, 1963)—the principal loadings on PC1 were the ratio variables: HWI, relative tail length, and relative tarsus length. The size variables—toe length and bill volume—were the principal loadings on PC2 (Table 3). Some of the genera formed clear clusters in morphological space (*Zosterops*, *Symposiachrus*, and *Rhipidura rufifrons* and *Rhipidura cockerelli*), while others were less distinct (*Monarcha* and *Myiagra*; Figure 6). Using the knn algorithm, we were able to correctly assign species identity 78% of the time (standard deviation = 0.07), and subspecies identity 60% of the time (standard deviation = 0.08) based on morphology.

**TABLE 2:** Morphology principal component analysis

Principal Component Axis	PC1	PC2	PC3	PC4	PC5
Proportion of Variance Explained	0.37	0.33	0.19	0.06	0.05
Cumulative Variance Explained	0.37	0.70	0.88	0.95	1

**TABLE 3:** Principal component analysis loadings

Trait	PC1	PC2	PC3	PC4	PC5
Hand‐wing index	−0.63	0.09	−0.32	−0.66	−0.25
Relative tail length	−0.62	0.18	−0.26	0.71	0.12
Relative tarsus length	0.44	0.25	−0.72	0.14	−0.45
Toe length	0.06	−0.63	−0.54	−0.08	0.55
Bill volume	−0.14	−0.71	0.12	0.21	−0.64

### 3.3 Correlating morphology and behavior

**FIGURE 7:**
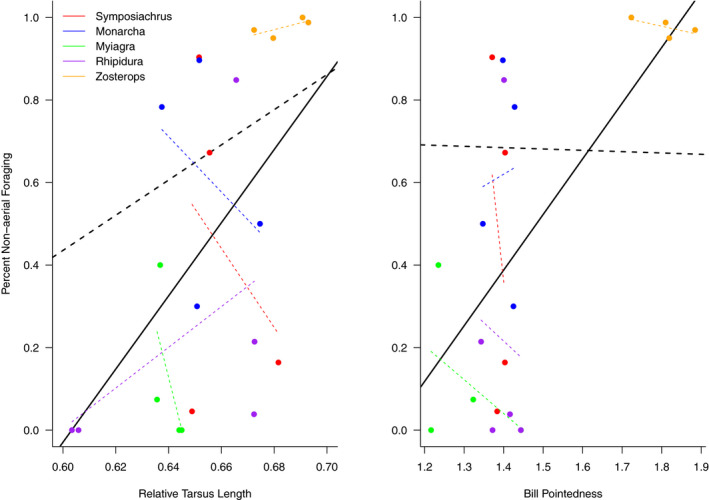
Phylogenetic constraint drives the relationship between morphology and behavior. Increased relative tarsus length (the ratio of tarsus length to wing length) and increased bill pointedness (bill length divided by bill width) have been associated with increased ground foraging in tyrannid flycatchers (Fitzpatrick, 1985). In our data, we recover a positive relationship between both tarsus length and bill pointedness and non‐aerial foraging (solid black lines). However, this relationship is not significant after accounting for phylogenetic nonindependence in the data (dashed black lines). This reflects the idiosyncratic relationships at smaller taxonomic scales (colored lines represent within‐genus relationships) despite a broad correlation between behavior and taxonomy across groups (Figure 1)

Results from the CCA indicated that morphology and foraging behavior of all taxa across islands are correlated, though this relationship is marginally significant (*n* = 21, Pillai's trace = 1.26, *p* = .05). However, when phylogenetic relatedness was controlled for in the pCCA, this relationship was not significant (canonical correlation 1, *p* = .66, *λ* = 0.74). Similarly, relative tarsus length and bill pointedness were significantly positively related with the proportion of non‐aerial foraging (*r* ^2^ = .25 and .35, and *p* = .01 and *p* = .002, respectively; Figure 7). However, as with the CCA, once phylogeny was controlled for, these relationships were no longer significant (*p* = .36 and *p = *.98, respectively).

## 4 DISCUSSION

While morphology has the potential to serve as a powerful tool for understanding how species partition niche space (Gomez, Bravo, Brumfield, Tello, & Cadena, 2010; Ricklefs & Travis, 1980), this is based on the presumption that differences in morphology coevolve with changes in behavior. In birds, testing this relationship has often taken the form of correlating functional trait measurements with ordinations of multivariate representations of complex foraging behaviors, but these efforts have focused on continental systems (Botero‐Delgadillo & Bayly, 2012; Fitzpatrick, 1985; Miles & Ricklefs, 1984), have often explored correlations between distantly related taxa (Miles & Ricklefs, 1984), and have not typically accounted for phylogenetic relatedness among taxa. Our results suggest morphology and behavior are correlated in this island system, but only at broad taxonomic scales; within‐clade relationships reveal a more complex pattern.

Small scale shifts in the relationship between morphology and behavior may be driven by behavioral plasticity, associated with the ecological interactions inherent in mixed‐species flock participation. The flocks characterized here appear to be structured largely by complementary rather than supplemental benefits, *sensu* Goodale et al. (2020). We do not note any examples of convergence in plumage or call that might suggest the flocks were shaped by supplementary benefits. In *Zosterops‐*led flocks in the Seychelles, there is evidence of “imitative foraging” in which other species match *Zosterops* in foraging maneuver and location (Greig‐Smith, 1978). While we do not have foraging data for species outside of flocks, we do not find evidence of “imitative foraging” (i.e., supplementary foraging benefits) across islands. Rather, the Solomons flocks appear more akin to the “small insectivore alliances” in New Guinea (Bell, 1983). In the Solomons, the constituent species appear to follow *Zosterops* (Table 1), taking advantage of their boisterous disturbance of the canopy to capture prey. This dynamic is analogous to New Guinea flocks forming around the gregarious *Gerygone* spp. (Bell, 1983), and one that is found across a range of taxa and systems (Goodale & Beauchamp, 2010; Satischandra et al., 2007; Sridhar & Shanker, 2013, 2014). This sets the flocks of the Solomons apart from *Zosterops‐*led flocks in Sri Lanka where there is limited evidence of other taxa targeting prey disturbed by *Zosterops* (Partridge & Ashcroft, 1976). Additional study of the ecologies of the Solomons flocks is necessary to understand how they are structured, the propensity of species to join flocks, and the extent to which species alter their behaviors when they join a mixed‐species flock. Ecological interactions inherent in mixed‐species flocks can alter foraging behavior in myriad ways including shifts in the food resources targeted (Greig‐Smith, 1978) and changes in where and how foraging occurs (Chen & Hsieh, 2002; Farine & Milburn, 2013; Hino, 2000). The behavioral plasticity of many species upon joining mixed‐species flocks thus results in dynamic relationships between morphology and behavior that are contingent on biotic interactions.

In addition to flocking‐induced behavioral shifts, when species colonize islands and encounter novel and relatively depauperate systems, they are likely to adapt rapidly to a suite of both abiotic and biotic pressures (Grant & Grant, 2002; Lescak et al., 2015). For example, island colonization has been associated with ecological shifts in birds of the Southwest Pacific, including shifts in microhabitat use and changes in foraging technique (Diamond, 1970). Biotic pressures may be particularly powerful when species' life histories include complex interspecific interactions, for example foraging in mixed‐species flocks.

Our results suggest that the relationship between behavior and morphology has an important phylogenetic component, and we do not find evidence that congeneric relationships between morphology and behavior are consistent with large‐scale patterns (Figures 1 and 7). When the phylogenetic relatedness of species is not accounted for, our multivariate canonical correlation suggests that morphology is correlated with foraging behavior across a wide range of taxa (*p* = .05). However, this correlation breaks down after accounting for phylogenetic relatedness (*p* = .66), suggesting that the relationship between morphology and behavior has an important phylogenetic component (also reflected in the high pCCA *λ* = 0.74).

The importance of phylogenetic scale for the relationship between morphology and behavior is reflected in the two traits that we examined individually. Relative tarsus length and bill pointedness are significantly correlated with percent of non‐aerial foraging (*p* = .01 and *p = *.002, respectively). This is in line with similar findings for distantly related ground foragers within the Tyrannidae in the New World tropics (Fitzpatrick, 1985), suggesting that similar morphologies may be well suited to non‐aerial foraging, despite the extreme differences in ground‐dwelling and arboreal species morphologies. However, as with the multivariate analyses, these bivariate relationships break down once phylogenetic relatedness has been accounted for in the model. We find that the within‐genus bivariate relationships between morphology and behavior do not appear to match the among‐genus relationships (Figure 7). While we interpret these findings cautiously, due to our limited sample sizes, we suggest two possible mechanisms for this decoupling of morphology and behavior at small scales. First, it is possible that behavioral plasticity associated with flocking differs among taxa. Second, the behaviors of these species have shifted across islands, and morphology may have changed to accommodate local adaptation in different ways within different genera. For example, in some genera, species that tend toward increased aerial foraging have reduced tarsus length or bill pointedness, while in other genera, species that tend toward increased aerial foraging have increased tarsus length or bill pointedness (Figure 7). These idiosyncratic patterns at small taxonomic scales further support the idea that while behavior and morphology may be correlated at broad taxonomic scales, within smaller taxonomic groups there is variation in the relationship between more subtle changes in morphology and behavior. Understanding the scale at which morphology consistently predicts behavior across taxa will inform understanding of the relationship between local adaptation and macroevolutionary morphological patterns.

The ability to distinguish between taxa using morphology reflects rapid changes in morphology, potentially indicating a role for selection. Strong selection on island populations can result in rapid morphological and behavioral shifts. Idiosyncratic relationships between rapid changes in morphology and behavior at small scales may produce intrageneric patterns that do not correspond to larger scale relationships. This could temporarily decouple morphology and behavior. Similarly, the increased strength of intraspecific competition relative to interspecific competition on relatively depauperate islands may result in increased niche breadth, with concomitant increases in intrapopulation morphological variability (i.e., the niche variation hypothesis; Van Valen, 1965). This broadening of the population niche can result from either the expansion of each individual's niche, or increasing variation among the niches of individuals within the population (Bolnick et al., 2010). While short‐term behavioral plasticity may decouple behavior and morphology, it also has the potential to be an important mechanism driving evolutionary change (Price, Qvarnström, & Irwin, 2003). This enhanced within‐taxon morphological variation is unlikely to obscure correlated morphologies and behaviors at coarse taxonomic scales (e.g., among different families). However, within‐taxon variation could be capable of breaking down the correlation between morphology and behavior within smaller taxonomic groups (e.g., correlations between the shifts in morphology and behavior between populations of the same species on different islands).

Our results add complexity to the long‐standing observation that species distributions and ecologies shift depending on the presence of other—typically closely related—species (Diamond, 1975b). Collectively, the evidence of a role for phylogenetic history and idiosyncratic shifts in the relationship between morphology and behavior of taxa across islands reflect interactions between abiotic, biological, and historical contingencies. The complexity of localized changes in behavior and morphology represents both a challenge to the use of morphology as a proxy for behavior, and an exciting avenue of future research into the relationship between ecological and morphological shifts at the species and community levels.

## CONFLICT OF INTEREST

The authors have no conflicts of interest to declare.

## AUTHOR CONTRIBUTIONS

**Brian C. Weeks:** Conceptualization (lead); formal analysis (lead); funding acquisition (lead); investigation (lead); methodology (lead); visualization (lead); writing – original draft (lead); writing – review & editing (lead). **Shahid Naeem:** Conceptualization (supporting); resources (supporting); supervision (supporting); writing – review & editing (supporting). **Benjamin M. Winger:** Writing – review & editing (supporting). **Joel Cracraft:** Conceptualization (supporting); resources (supporting); supervision (supporting); writing – review & editing (supporting).

## Supporting information

Appendix S1

Appendix S2

Appendix S3

## Data Availability

All morphological and behavioral data are available in Appendices S1 and S2.
