# Supplementary material for: The relationship between morphology and behavior in mixed‐species flocks of island birds
Source: Ecol Evol. 2020 Sep 25;10(19):10593–606. doi: 10.1002/ece3.6714 (PMC7548193; doi:10.1002/ece3.6714)
Supplement: Supplementary file 3 — Appendix S3 [file ECE3-10-10593-s003.docx]

**Weeks, B.C., et al. The relationship between morphology and behavior in mixed-species flocks of island birds.**

**Appendix 3. No evidence that different sample sizes across islands biased shifts in foraging stratum location or maneuver.**

In order to test the impact of variable sample sizes across islands on our characterization of foraging behavior, we randomly subsampled foraging observations from islands with higher numbers of observations, such that the same number of observations was used to characterize foraging on each island. We then calculated the percent foraging that occurred in different strata (Appendix Figure 1), and the percent of foraging comprised of each type of prey capture maneuver (Appendix Figure 2). The sample sizes are the minimum sampling on an island for each genus

*Appendix Figure 1.* Distributions of foraging moves through the canopy, estimated with consistent sample sizes across islands. Sample sizes are noted above each column, as in Figure 3.

*Appendix Figure 2:* Proportion of foraging maneuver type across islands, estimated with consistent sample sizes across islands. Sample sizes match Appendix Figure 1.
